# Generation of Human‐Induced Pluripotent Stem Cells From Anterior Cruciate Ligament

**DOI:** 10.1002/jor.24493

**Published:** 2019-10-25

**Authors:** Steven Woods, Nicola Bates, Sara L. Dunn, Ferdinand Serracino‐Inglott, Tim E. Hardingham, Susan J. Kimber

**Affiliations:** ^1^ Division of Cell Matrix Biology and Regenerative Medicine, School of Biological Sciences University of Manchester Michael Smith Building, Oxford Rd Manchester M13 9PT United Kingdom; ^2^ Division of Cell‐Matrix Biology and Regenerative Medicine, Wellcome Trust Centre for Cell‐Matrix Research, Faculty of Biology, Medicine and Health, School of Biological Sciences University of Manchester Manchester United Kingdom; ^3^ Department of Vascular Surgery Central Manchester NHS Foundation Trust Manchester United Kingdom

**Keywords:** anterior cruciate ligament, human induced pluripotent stem cells, reprogramming, ligament differentiation, tissue engineering

## Abstract

Human‐induced pluripotent stem cells (hiPSCs) are reprogrammed somatic cells and are an excellent cell source for tissue engineering applications, disease modeling, and for understanding human development. HiPSC lines have now been generated from a diverse range of somatic cell types and have been reported to retain an epigenetic memory of their somatic origin. To date, the reprogramming of a true ligament has not been reported. The aim of this study is to generate iPSCs from human anterior cruciate ligament (ACL) cells. ACL cells from three above‐knee amputation donors, with donor matched dermal fibroblasts (DFs) were tested for reprogramming using an existing DF reprogramming protocol. ACL cells were, however, more sensitive than donor matched DF to transforming growth factor‐β (TGF‐β); displaying marked contraction, increased proliferation and increased *TNC* and *COMP* expression in vitro, which hindered reprogramming to iPSCs. Modification of the protocol by scoring the cell monolayer or by removal of TGF‐β during ACL reprogramming resulted in emerging colonies being easier to identify and extract, increasing reprogramming efficiency. Following 30 passages in culture, the generated ACL derived iPSCs displayed pluripotency markers, normal karyotype and can successfully differentiate to cells of the three embryonic germ layers. This study illustrates it is possible to generate hiPSCs from ligament and identifies optimized ligament reprogramming conditions. ACL derived iPSCs may provide a promising cell source for ligament and related tissue engineering applications. © 2019 The Authors. *Journal of Orthopaedic Research*® published by Wiley Periodicals, Inc. on behalf of Orthopaedic Research Society J Orthop Res 38:92–104, 2020

Human‐induced pluripotent stem cells (hiPSC) are reprogrammed somatic cells, first generated in human cells by overexpression of *OCT4, KLF4, SOX2*, and *c‐MYC* in dermal fibroblasts (DFs).^1^, ^2^ HiPSCs are similar to human embryonic stem cells (hESC); they are capable of self‐renewal and differentiation to multiple cell types derived from all three embryonic germ layers, making them an ideal cell source for tissue engineering and in vitro disease modeling.

HiPSCs have been generated from a wide range of somatic cell types, as well as DFs,^1^, ^2^ these include peripheral blood mononuclear cells (PBMCs),^3^ squamous epithelial cells from urine,^4^ cord blood,^5^ keratinocytes,^6^ extra‐embryonic tissues,^7^ hepatocytes,^8^ pancreatic islet beta cells,^9^ synovial cells,^10^, ^11^ wisdom teeth mesenchymal stromal cells,^12^ periodontal ligament cells,^13^ and articular chondrocytes.^14^ Generation of iPSCs from a true or articular ligament (a ligament connecting bone to bone) has not been reported.

iPSCs have been reported to retain an epigenetic memory embedded within partially retained chromatin structure^9^ and with DNA methylation,^15^, ^16^ gene expression,^17^ and differentiation being skewed towards their parental cell type. Skewed differentiation has previously been demonstrated for the hepatic,^18^ haematopoietic^19^, ^20^ and pancreatic lineages.^9^

Ligament and tendon have limited regeneration ability. PSCs are slowly becoming recognized as a potential source of therapeutic cells for ligament and tendon repair.^21^, ^22^ However their exploitation in this field has lagged behind the differentiation of such cells for cartilage and bone repair.^23^, ^24^, ^25^ Although there are few studies addressing tendon differentiation from PSC, some pioneering papers have emerged. For instance, tenogenic differentiation of PSCs has been achieved through rolling cell sheets derived from PSC‐derived MSC/connective tissue progenitor intermediates^21^, ^26^ and also driven directly from PSCs using BMP12 and BMP13.^22^

The aim of this study was to generate iPSCs from the anterior cruciate ligament (ACL). Doing so will provide an additional cell source for iPSCs. In addition, due to the reported epigenetic memory of iPSCs, human ACL‐iPSCs may be more amenable to differentiation to skeletal tissues, of common mesodermal origin. This will thus provide an ideal cell population to study human ligament development and for tissue engineering applications, such as generating cell‐based therapies for the treatment of ACL rupture.

Here we report the first reprogramming of ACL to hiPSCs though which we found critical differences in requirements from DF reprogramming.

## METHODS

### Isolation of DF and ACL Cells

The use of human material for this study was approved by the UK Integrated Research Application System (IRAS 114697) and University Ethics Committee. Patients undergoing above the knee amputation with peripheral vascular disease and no history of the joint disease gave informed consent to participate in this study.

For isolation of DF cells, a piece of skin ~1 cm^2^ was dissected from an area with no clinical sign of vascular disease near to the knee and washed three times with phosphate‐buffered saline (PBS) containing 100 U/ml penicillin, 100 μg/ml streptomycin, and 2.5 µg/ml amphotericin B. A scalpel and forceps were used to remove the subcutaneous fat. Skin was then cut into ~1 mm pieces, followed by treatment with collagenase type I (12 mg collagenase in 4 ml medium/g of tissue, C0130; Sigma‐Aldrich (Cambridge, UK), sterilized by passing through a 0.2 µm filter) at 37°C for 3 h. After this smaller pieces remained and these were allowed to settle in 15 ml tubes and washed with fresh Dulbecco's modified Eagle's medium (DMEM) + 10% fetal calf serum (FCS). These pieces were then placed into a T75 flask (Corning, Amsterdam, Netherlands) and allowed to outgrow in DMEM + 10%FCS, 2 mM glutamine, 100 U/ml penicillin, 100 μg/ml streptomycin, 2.5 µg/ml amphotericin B, and 50 μg/ml ascorbic acid. Outgrowth was observed within 10 days and cells were passaged 1:4 when 50% confluent (within 28 days) using TrypLE™ (Thermo Fisher Scientific, Altrincham, UK). Cells continued to be passaged 1:4 when 70–80% confluent thereafter, until passage 3 when reprogramming took place.

For isolation of ACL cells, a small piece of ACL was remove using scalpel and forceps and washed three times with PBS containing amphotericin and penicillin/streptomycin, ACL was then cut into ~1 mm pieces, followed by treatment with collagenase type I (12 mg collagenase in 4 ml medium/g of tissue, sterilized by passing through a 0.2 µm filter, C0130; Sigma‐Aldrich) at 37°C for 3 h. Using a 70 µm cell strainer (FB35181; Fisher Scientific, Altrincham, UK) any non‐digested tissue was removed. Cells which passed through the cell strainer were then centrifuged at 400 g for 4 min, the supernatant removed, cells washed in DMEM + 10% FCS. The cells were then plated into a T75 flask and allowed to grow in DMEM + 10% FCS, 2 mM glutamine, 100 U/ml penicillin, 100 μg/ml streptomycin, 2.5 µg/ml amphotericin B, and 50 μg/ml ascorbic acid. Large groups of proliferating adherent cells were observed within 10 days and cells were passaged 1:4 when ~50% confluent (within 28 days) using TrypLE. Cells were then passaged 1:4 when 70–80% confluent thereafter, until passage 3 when reprogramming took place.

### Reprogramming of DF and ACL Cells to iPSCs

Reprogramming was performed on passage 3 DF and ACL cells. Three different reprogramming protocols were tested, these are detailed below and summarized in Table 1. Where necessary emerging colonies were stained for the pluripotency‐associated marker TRA‐1‐81 using StainAliveTM DyLightTM488 (09‐0069; Stemgent, Glasgow, UK).

**Table 1:** Protocols Tested for Reprogramming of DF and ACL Cells to iPSCs

Day −1	Day 0	Day 1, 3, and 5	Day 7	Day 8, 9, 10, and 11	Day 12	Day 13 Until Colony Isolation (Between Day 18 and 24)	Colony Isolation (Between Day 18 and 24)	Efficiency
Plate cells in 24‐well plate (DMEM + 10% FCS)	Cytotune™‐iPS 2.0 Sendai (MOI of 5:5:3)	Refresh medium (DMEM + 10% FCS)	Transfer to VTN coated six‐well plates (DMEM + 10% FCS)	Remove medium and replace with E8	Protocol 1: Remove medium and replace with E8	Remove medium and replace with E8	Transfer colonies to VTN‐coated plates, containing E8. Continue to grow on VTN coated plates in E8 medium.	DF: 0.066–0.385% ACL: not successful
					Protocol2: Score cell monolayer. Remove medium and replace with E8	Remove medium and replace with E8		DF: Not tested ACL: 0.005–0.02%
					Protocol 3: Remove medium and replace with E6 + 100 ng/ml FGF2	Remove medium and replace with E6 + 100 ng/ml FGF2		DF: 0.165% ACL: 0.04%

ACL, anterior cruciate ligament; DF, dermal fibroblast; DMEM, Dulbecco's modified Eagle's medium; FCS, fetal calf serum; FGF, fibroblast growth factor; iPSC, induced pluripotent stem cell; MOI, multiplicity of infection; VTN, vitronectin.

#### Reprogramming protocol#1 (with TGF‐β: successful for DF but not ACL cells)

Protocol#1 was based upon the supplier's protocol for the CytoTune™‐iPS 2.0 Sendai Reprogramming Kit (http://www.thermofisher.com). DF and ACL cells were subject to TrypLE and plated at 20,000–50,000 cells per well in a 24‐well plate and incubated overnight. Cells were then transduced using CytoTune™‐iPS 2.0 Sendai virus (A16517; Thermo Fisher Scientific) at an MOI of 5:5:3 (KOS [Klf4, Oct4, and Sox2] = 5: hc‐Myc = 5: hKlf4 = 3) in 200 μl DMEM + 10% FCS (day 0). The medium was refreshed on day 1, 3, and 5 after transduction. On day 7 after transduction cells were subject to TrypLE, re‐suspended in 8 ml DMEM + 10% FCS and transferred to fresh vitronectin (VTN) coated six‐well plates (10 μl truncated vitronectin recombinant human protein Thermo Fisher Scientific‐A14700 + 1 ml PBS per well for 1 h at room temperature) at a range of cell densities: 4, 2, 1, and 0.5 ml medium containing cell suspension, with additional DMEM + 10% FCS added to wells, to give minimum of 2 ml per well, cell count ranged from 12,500 to 100,000 cells per well on day 7. On day 8 medium was switched from DMEM + 10% FCS to Essential 8 (E8) (Thermo Fisher Scientific), and then refreshed daily until colonies containing more than 50 cells with pluripotent morphology or positive stain for TRA‐1‐81 (StainAliveTM DyLightTM488, 09‐0069; Stemgent) were isolated using a pulled glass pipette (typically day 18–24 after transduction). Colonies were transferred to fresh VTN coated six‐well plates containing E8 when large enough to isolate. Colonies were maintained as PSCs in E8 thereafter.

#### Reprogramming protocol#2 (with TGF‐β and scoring on day 12: low efficiency for ACL cells)

Reprogramming was performed as described for protocol#1, with the addition of scoring through the cell monolayer in a criss–cross pattern of around 20 scores/well on day 12 after transduction. Scoring was performed using the edge of a pulled glass pipette, each score was around 20 mm in length. This scoring modification prevented ACL cells from rolling up into one sheet and gave developing colonies space to grow.

#### Reprogramming protocol#3 (absence of TGF‐β from day 8 after transduction: Successful for HDF and ACL cells)

Reprogramming was performed as described for protocol#1, but without TGF‐β from day 8 until colony isolation. On day 8 after the transduction medium was switched from DMEM + 10% FCS to Essential 6 (Thermo Fisher Scientific) + 100 ng/ml FGF2, colonies were switched back to E8 following isolation. The removal of TGF‐β prevented ACL cells from rolling up into a single sheet and gave developing PSC colonies space to grow.

Reprogramming efficiency was calculated by dividing the number of identified pluripotent colonies by the number of cells transduced. Not all identified pluripotent colonies were taken forward into established lines, but all were still taken into account when calculating efficiency.

### Culture of DF‐iPSC and ACL‐iPSC

Pluripotent iPSCs were cultured at 37°C 5% CO_2_ on VTN coated six‐well plates in E8 (Thermo Fisher Scientific) as colonies and passaged every 4–7 days using 0.5 mM ethylenediaminetetraacetic acid (EDTA) (15575020; Thermo Fisher Scientific).

### Generation of Embryoid Bodies (EBs)

Pluripotent DF‐iPSC and ACL‐iPSC colonies were divided into squares by scoring using a pulled glass pipette. They were detached by pipetting, transferred to non‐adherent tissue culture plates containing DMEM + 10% FCS and cultured for 10 days to form EBs. EBs were then transferred to FCS coated 24‐well plates and cultured for a further 5 days to allow outgrowth before staining.

### Immunostaining

Established pluripotent (beyond passage 15) DF‐iPSCs and ACL‐iPSCs were seeded into 24‐well plates and grown for 4 days. Pluripotent cells and outgrown EBs were fixed with 4% paraformaldehyde in PBS for 10 min. Cells were then incubated with primary antibodies against markers of pluripotency; NANOG (1:400, cat. no. 4903; Cell Signaling Technology, London, UK), OCT‐4 (1:100, cat. no. 611202; BD Biosciences, Oxford, UK), SOX2 (1:400, cat. no. 3579; Cell Signaling Technology), SSEA‐3 (1:200, cat. no MAB1434; R&D Systems, Abingdon, UK), SSEA‐4 (1:200, cat. no MAB1435; R&D Systems), TRA‐1‐60 (1:200, cat. no. Ab16288; Abcam, Cambridge, UK), TRA‐1‐81 (1:200, cat. no. Ab16289; Abcam), marker of early differentiation; SSEA‐1 (1:200, cat. no. MAB2155; R&D Systems), marker of mesoderm; α‐smooth muscle actin (αSMA) (1:100, cat. no. MAB1420; R&D Systems), marker of endoderm; GATA6 (1:1600, cat. no. 5851; Cell Signaling Technology) and marker of ectoderm; Neurofilament (1:100, cat. no. 2837; Cell Signaling Technology), in the presence of 1% goat serum, followed by Alexa Fluor secondary antibodies (1:200; Thermo Fisher Scientific) and nuclei stained using 4′,6‐diamidino‐2‐phenylindole (DAPI) (cat no. D1306; Thermo Fisher Scientific). Images were captured using BX51 fluorescence microscope (Olympus, Southend‐on‐Sea, UK).

### Karyotyping

Pluripotent iPSCs were karyotyped as previously described.^27^

### Generation of iPSC‐MCs (iPSC‐Derived Mesenchymal Cells) From iPSCs

Colonies of iPSCs (between passage 15 and 20) were passaged using EDTA in order to obtain ~10 colonies per well of a six‐well plate. Four days after plating colonies reached ~500–800 μm in diameter (day 0), the medium was switched from E8 (pluripotent maintenance) to MesenPRO RS™ (mesenchymal stem cell maintenance medium; Thermo Fisher Scientific), MesenPRO RS™ was refreshed every 2 days until day 7. On day 7 cells were passaged to 0.1% gelatine coated T75 using TrypLE, (equivalent to a 1:7.5 split ratio or ~0.5 × 10^6^ to 1 × 10^6^ cells per T75), termed P0. MesenPRO RS™ was then refreshed every 3 days until day 14. On day 14 cells were passaged 1:8 to T75 (tissue culture plastic), termed P1. Mesenchymal cells (iPSC‐MCs) were then maintained in MesenPRO RS™ on tissue culture plastic and passaged 1:6 when ~80% confluent using TrypLE. TGF‐β stimulation was performed using 10 ng/ml TGFβ3 on passage 3 iPSC‐MCs in MesenPRO RS™ for 7 days.

### Gene Expression Analysis

Total RNA was extracted using RNeasy Mini kit (Qiagen, Manchester, UK). Reverse‐transcription was performed using M‐MLV reverse‐transcriptase (Promega, Southampton, UK). Real‐time polymerase chain reaction (PCR) for gene expression was assessed using gene‐specific primers (Table 2) and Power SYBR Green PCR Master Mix (Applied Biosystems, Edinburgh, UK) with a Bio‐Rad C1000 touch thermocycler (Watford, UK). Gene expression was normalized to *GAPDH*.

**Table 2:** Primers Used for Real‐Time Polymerase Chain Reaction (PCR) Analysis

Genes	Forward	Reverse
GAPDH	ATGGGGAAGGTGAAGGTCG	TAAAAGCAGCCCTGGTGACC
TNC	TCTCTGCACATAGTGAAAAACAATACC	TCAAGGCAGTGGTGTCTGTGA
SCX	CGAGAACACCCAGCCCAAAC	ACCTCCCCCAGCAGCGTCT
COMP	AGGACAACTGCGTGACTGTG	GTGTCCTTTTGGTCGTCGTT
DCN	CTCTGCTGTTGACAATGGCTCTCT	TGGATGGCTGTATCTCCCAGTACT
COL1A1	CTGGTGATGCTGGTCCTGTTG	CCTTGGGGTTCTTGCTGATGT
POU5F1 (OCT4)	AGACCATCTGCCGCTTTGAG	GCAAGGGCCGCAGCTTA
NANOG	GGCTCTGTTTTGCTATATCCCCTAA	CATTACGATGCAGCAAATACAAGA
SOX2	AACCAGCGCATGGACAGTTAC	TGGTCCTGCATCATGCTGTAG
CD44	ACGTGGAGAAAAATGGTCG	TTGAAAGCCTTGCAGAGGT
NT5E (CD73)	CTCCTCTCAATCATGCCGCT	TGGATTCCATTGTTGCGTTCA
MCAM (CD146)	CAGGGAAGCAGGAGATCACG	CAGGAGGCCCATCTCTTCTG
ALCAM (CD166)	CTGGCAGTGGAAGCGTCATA	TCTCTGTTTTCATTAGCAGAGAC

### Statistical Analysis

Statistical differences were calculated by either one‐way or two‐way analysis of variance (ANOVA) followed by Bonferonni multiple comparison adjustments, as indicated within figure legends. In all cases *p < 0.05, **p < 0.01, ***p < 0.001, and non‐significant *p* > 0.05.

## RESULTS

### ACL Cells Can be Partially Reprogrammed Using a Protocol Optimized for DFs (Protocol#1)

**Figure 1:**
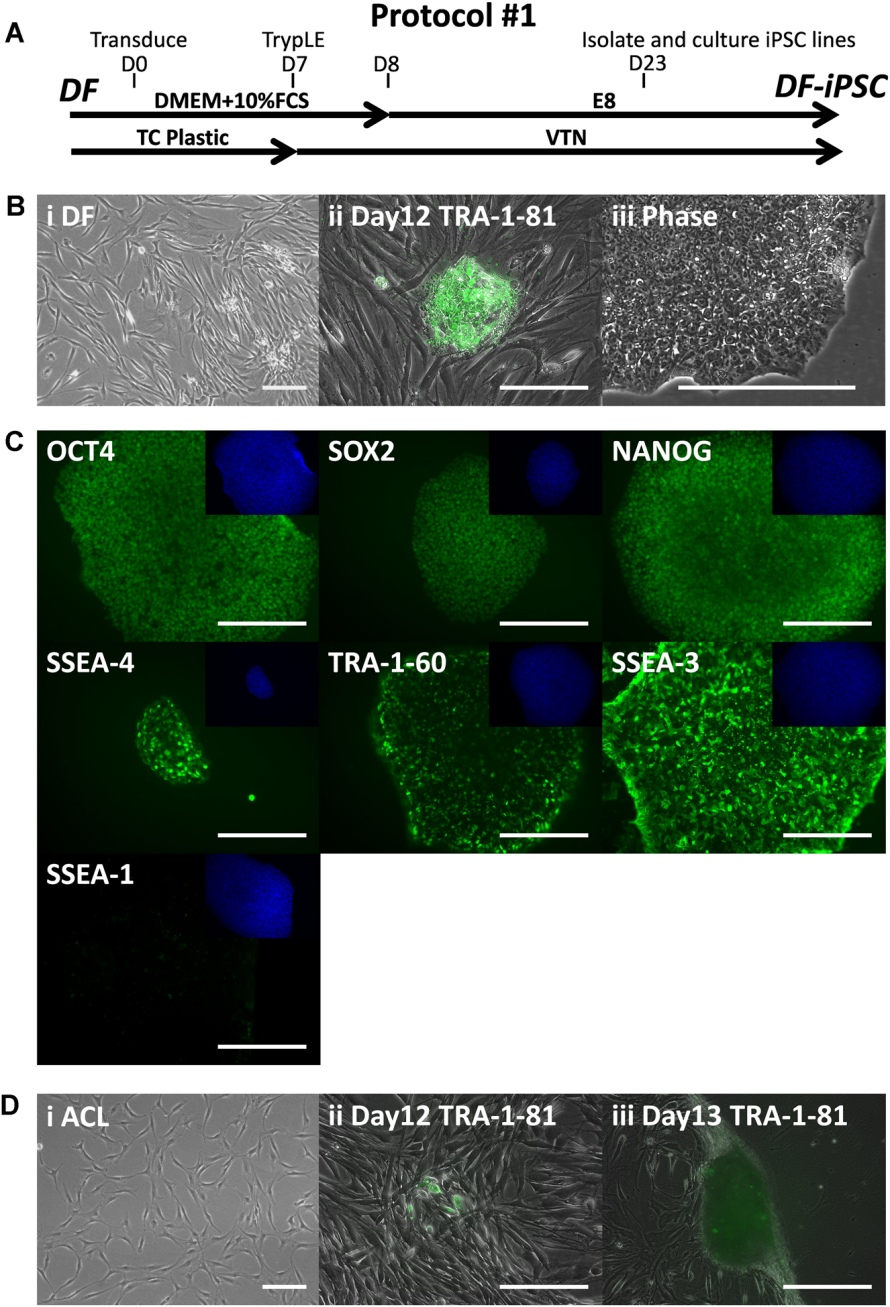
Anterior cruciate ligament cells can be partially reprogrammed using a protocol optimized for dermal fibroblasts (protocol#1). (A) Isolated donor‐matched dermal fibroblast (DF) and anterior cruciate ligament (ACL) were subject to reprogramming protocol#1. (B) Successful reprogramming of DF to DF‐iPSC‐SW156A. (Bi) Isolated DF growing in monolayer in Dulbecco's modified Eagle's medium (DMEM) + 10% fetal calf serum (FCS), (Bii) TRA‐1‐81 positive (StainAliveTM) induced pluripotent stem cell (iPSC) colony emerging between DF. (Biii) Phase‐contrast image of isolated DF‐iPSC‐SW156A. (C) Positive antibody staining of established passage 22 DF‐iPSC‐SW156A for the pluripotency‐associated markers, OCT‐4, SOX2, NANOG, SSEA‐4, TRA‐1‐60, SSEA‐3, and lack of early differentiation marker SSEA‐1. 4′,6‐diamidino‐2‐phenylindole (DAPI) nuclear staining shown in the top right quadrant for each. For DF reprogramming 33 pluripotent colonies were identified from reprogramming of 50,000 cells representing the efficiency of 0.066%, six colonies were grown into established lines. (D) Unsuccessful reprogramming of ACL. (Di) Isolated ACL cells, (Dii) TRA‐1‐81 positive (StainAlive™) cells emerging on top of ACL cells on day 12, (Diii) ACL spontaneously contracting into ligament‐like structure, TRA‐1‐81 positive cell became trapped and unable to form a colony, therefore were impossible to isolate. Scale bars represent 200 μm. [Color figure can be viewed at wileyonlinelibrary.com]

Donor matched DF and ACL reprogramming was initially attempted using the manufacturer protocol for CytoTune™‐iPS 2.0 Sendai Reprogramming Kit, which had previously been successfully used for DF reprogramming in our laboratory (Fig. 1A). As expected, DF was successfully reprogrammed to DF‐iPSC (DF‐iPSC‐SW156) (Fig. 1B) using Sendai virus containing three reprogramming plasmid vectors (carrying *OCT4, C‐MYC, KLF4*, and *SOX‐2*). After picking colonies and expansion, the cells showed the characteristic PSC morphology of large nucleus, prominent nucleoli, and high nucleus to cytoplasm ratio (Fig. 1B) and were demonstrated to express markers of pluripotency (Fig. 1C). For ACL reprogramming, potential colonies with expected cell morphology and positive for pluripotency marker TRA‐1‐81 emerged by day 12 after exposure to the Sendai virus (Fig. 1D). However, unlike after DF reprogramming, the potential colonies grew on top of the remaining ACL somatic cells rather than on the VTN substrate between the somatic cells (Fig. 1D). On day 13 (13 days after exposure to Sendai virus and 5 days after transfer to E8 medium) the layer of ACL cells rolled up into a structure reminiscent of a ligament, trapping TRA‐1‐81 positive cells (Fig. 1D), and making colony isolation from ACL impossible. At lower initial seeding density (12,500 cells per well), rolling‐up was delayed, however potential pluripotent colonies still grew on top of the remaining ACL somatic cells and rolling‐up still then occurred when colony isolation was attempted, meaning colony isolation at the lower cell density was also impossible.

### ACL Cells Can be Reprogrammed to hiPSC (Protocol#2)

**Figure 2:**
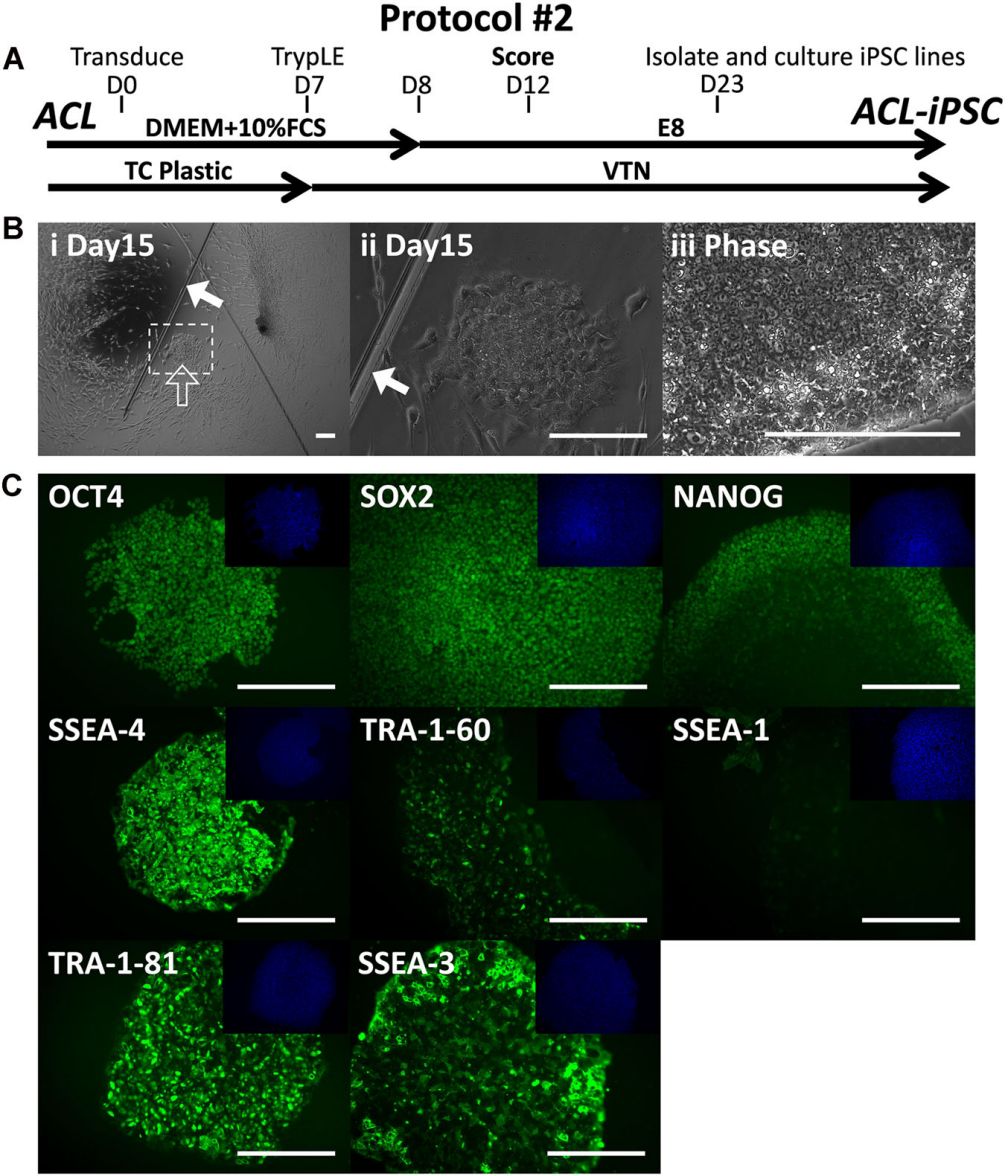
Generation of ACL‐iPSC‐SW157A from anterior cruciate ligament (ACL) by scoring the culture on day 12 (protocol#2). (A) Isolated ACL cells were subject to the reprogramming protocol#2. (B) Successful reprogramming of ACL to ACL‐iPSC‐SW157A. (Bi–Bii) scoring with a pulled glass pipette (filled arrow) prevented the entire well from rolling up and allows putative induced pluripotent stem cell (iPSC) colonies to grow in the space provided (open arrow), (Biii) phase contrast image of isolated ACL‐iPSC‐SW157A. (C) Positive antibody staining of established passage 22 ACL‐iPSC‐SW157A for the pluripotency‐associated markers OCT‐4, SOX2, NANOG, SSEA‐4, TRA‐1‐60, TRA‐1‐81, SSEA‐3, and lack of early differentiation marker SSEA‐1. 4′,6‐diamidino‐2‐phenylindole (DAPI) nuclear staining shown in the top right quadrant for each. Eight pluripotent colonies were identified (four grown into established lines) from reprogramming of 50,000 cells, representing the efficiency of 0.020%. Scale bars represent 200 μm. [Color figure can be viewed at wileyonlinelibrary.com]

Aiming to succeed in isolating the TRA‐1‐81 positive ACL‐derived iPSC colonies we applied a simple yet critical modification; scoring the cell monolayer with a pulled glass pipette in a criss‐cross pattern of around 20 scores on day 12 to prevent the entire cell culture from rolling up (protocol#2, Fig. 2A). Some retraction of cells was observed, but sufficient cells remained adherent in order to expect that some colonies would emerge. Indeed, potential iPSC colonies grew in the spaces generated by scoring (Fig. 2B), four clonal lines were successfully isolated between day 20 and day 30, grown to passage 15, and determined to be positive for pluripotency‐associated markers (Fig. 2C). These are the first reported hiPSC lines generated from ACL (ACL‐iPSC‐SW157A). ACL‐iPSCs have now been grown for 30 passages and retain PSC morphology and pluripotency‐associated marker expression.

### Generation of ACL‐iPSCs From a Second Donor

**Figure 3:**
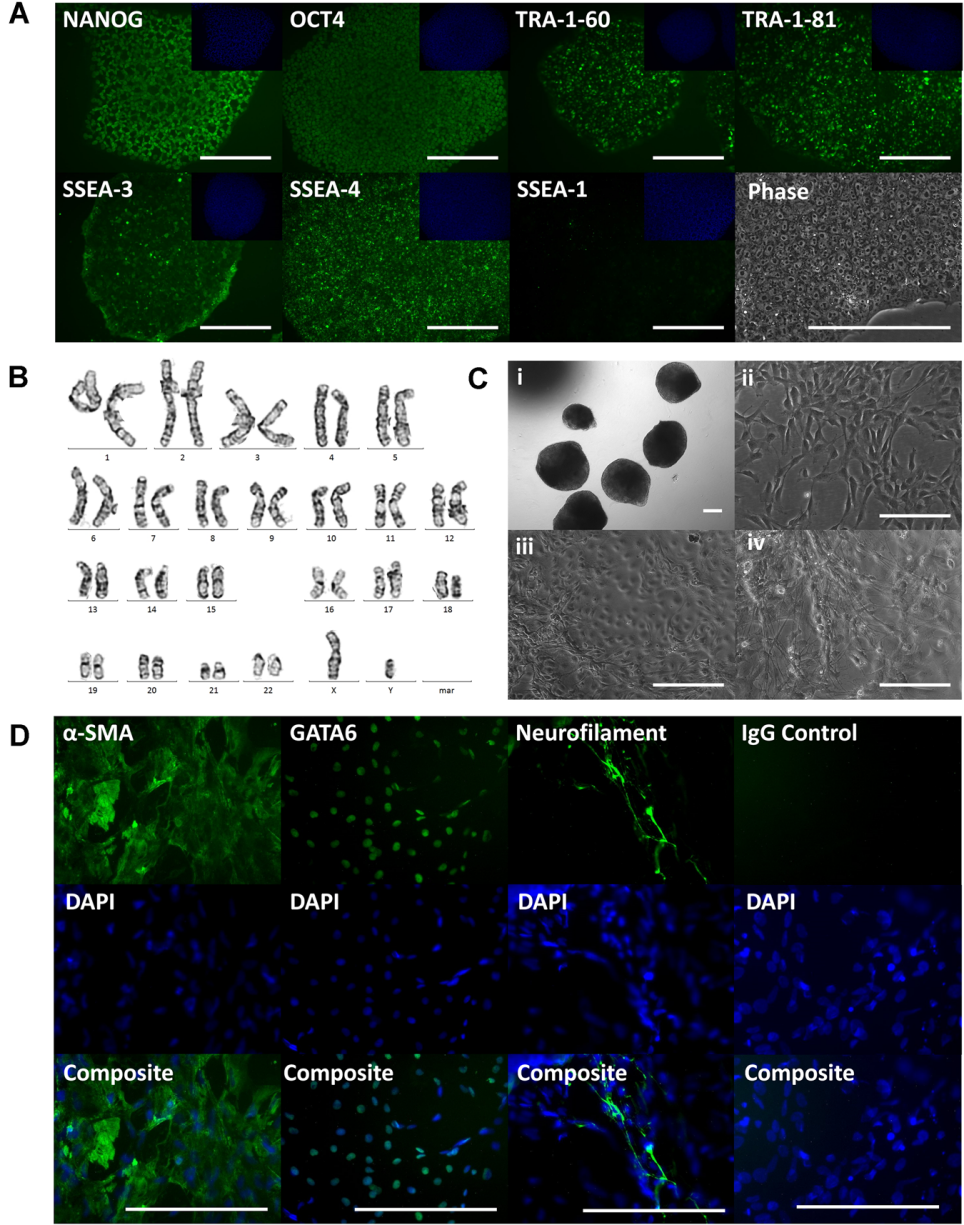
Generation and characterization of ACL‐iPSC‐SW163A (from a second donor using protocol#2). ACL‐iPSC‐SW163 derived using the scoring technique were grown to passage 20 (six colonies with pluripotent morphology were observed from 50,000 somatic cells giving an estimated efficiency of 0.012%). (A) ACL‐iPSC‐SW163A stained positive for antibodies to pluripotency markers NANOG, OCT‐4, TRA‐1‐60, TRA‐1‐81, SSEA‐3, SSEA‐4, with lack of early differentiation marker SSEA‐1. (B) ACL‐iPSC‐SW163 displayed a normal karyotype. (C) ACL‐iPSC can form embryoid bodies (EBs), (Ci) morphology of EBs at day 10, (Cii‐Civ) phase images displaying a range of morphologies of following EB outgrowth for 5 days. (D) Following outgrowth, EBs produced cells positive for antibodies to markers of mesoderm (α‐SMA), endoderm (GATA6), and ectoderm (Neurofilament). [Color figure can be viewed at wileyonlinelibrary.com]

Reprogramming with scoring modification (protocol#2) was used to generate another ACL‐iPSC line from a second donor (Fig. 3). The established ACL‐iPSC‐SW163 line displayed pluripotent morphology, was positive for pluripotency markers (Fig. 3A), had a normal karyotype (Fig. 3B) and was capable of forming EBs (Fig. 3Ci), which following outgrowth (Fig. 3Cii‐iv) produced cells positive for antibodies to markers of mesoderm (α‐SMA), endoderm (GATA6), and ectoderm (Neurofilament) (Fig. 3D). Although successful, this physical scoring protocol is very labor‐intensive and has a lower efficiency; 0.020% for the first ACL‐iPSC‐SW157 line (compared with 0.066% for donor matched DF) and only 0.012% for the second ACL‐iPSC‐SW163 line, which is lower than for all DF reprogramming performed in our laboratory to date (ranging from 0.066% to 0.405%).

### ACL Cells Proliferate and Form Aggregates in the Presence of TGF‐β

During our initial attempts at reprogramming, ACL cells but not DF formed a rolled‐up tissue 5 days after transition from DMEM + 10% FCS to E8 (Fig. 1D). We therefore aimed to identify a medium to prevent ACL cells from rolling‐up to form aggregates, avoiding the need for mechanical disruption and hence improve reprogramming efficiency of ACL.

**Figure 4:**
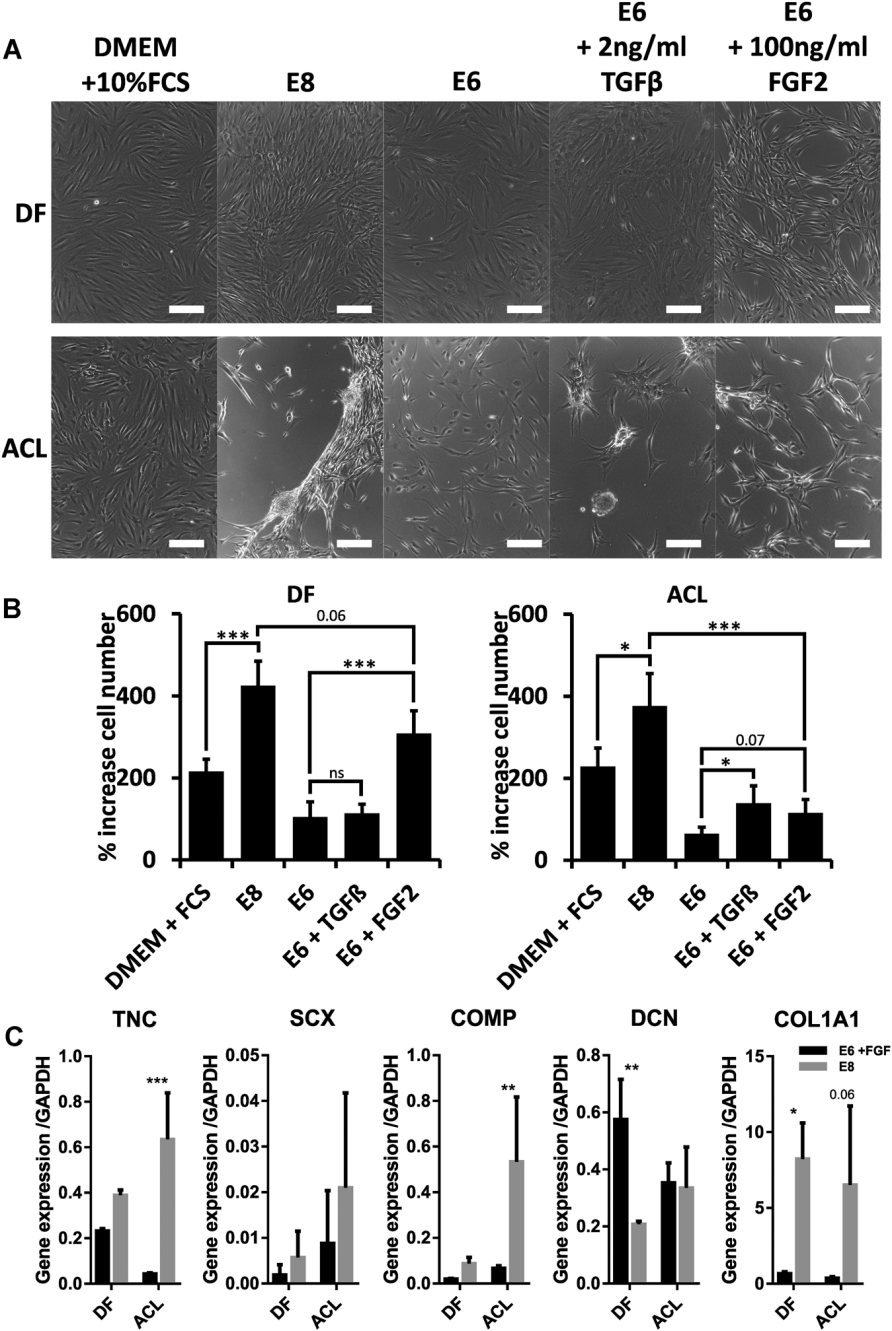
An anterior cruciate ligament (ACL) but not dermal fibroblast (DF) cells increase proliferation and form aggregates in response to transforming growth factor‐β (TGF‐β). Donor matched DF and ACL cells were plated at 10k cells/well in a 24‐well plate and grown in Dulbecco's modified Eagle's medium (DMEM) + 10% fetal calf serum (FCS) for 24 h. Cells were washed with phosphate‐buffered saline (PBS) and grown in E8, E6, E6 + 2 ng/ml TGF‐β1 or E6 + 100 ng/ml FGF2 for further 5 days. (A) Representative morphology of DF and ACL cultures after 5 days in different media, scale bars represent 200 μm. (B) Histograms showing percentage increase in cell number over a total of 6 days, data combined from two donors, three biological replicates per donor, error bars indicate standard deviation of biological replicates. Following dissociation with TrypLE, cells were counted using a Neubauer hemocytometer. Statistical differences calculated by one‐way analysis of variance (ANOVA) followed by Bonferonni multiple comparison adjustments (*p < 0.05, **p < 0.01, ***p < 0.001). (C) Quantitative real‐time polymerase chain reaction (qRT‐PCR) for TNC, SCX, COMP, DCN, and COL1A1 expression in DF and ACL cells after 5 days of culture in either E6 + FGF or E8. Statistical differences were calculated using one‐way ANOVA followed by Bonferonni multiple comparison adjustments (*p < 0.05, **p < 0.01, ***p < 0.001).

DF and ACL cells both increased proliferation following the transition from DMEM + 10% FCS to E8 (Fig. 4B). ACL cell but not DF cell layers contracted to form aggregates in E8 (Fig. 4A), reminiscent of the monolayer contraction observed during reprogramming protocol#1 or #2 (Figs. 1 and 2). However, if instead cells were transferred to E6 medium, which is identical to E8 but lacks TGF‐β and FGF2, the previous increase in DF and ACL cell proliferation and the ACL cell aggregate formation, were not observed (Fig. 4A and B). E6 with the addition of just TGF‐β increased ACL, but not DF cell proliferation (Fig. 4B), and induced ACL but not DF cells to form aggregates, compared with unmodified E6 (Fig. 4A). Furthermore, ACL but not DF proliferation was significantly higher in E8 when compared with E6 + FGF2 (equivalent to E8 lacking TGF‐β).

Using qRT‐PCR we investigated the transcriptional response of DF and ACL cells to TGF‐β. DF and ACL displayed higher expression of *TNC, COMP*, and *COL1A1* in E8 than in E6 + FGF (equivalent to E8 lacking TGF‐β) (Fig. 4C). Consistent with the changes in morphology and proliferation, ACL cells expressed more *TNC* and *COMP* in response to TGF‐β than DF (Fig. 4C). These data indicated ACL cells have increased sensitivity and response to TGF‐β compared with donor matched DF cells.

### Enhanced ACL Reprogramming in the Absence of TGF‐β (Protocol#3)

**Figure 5:**
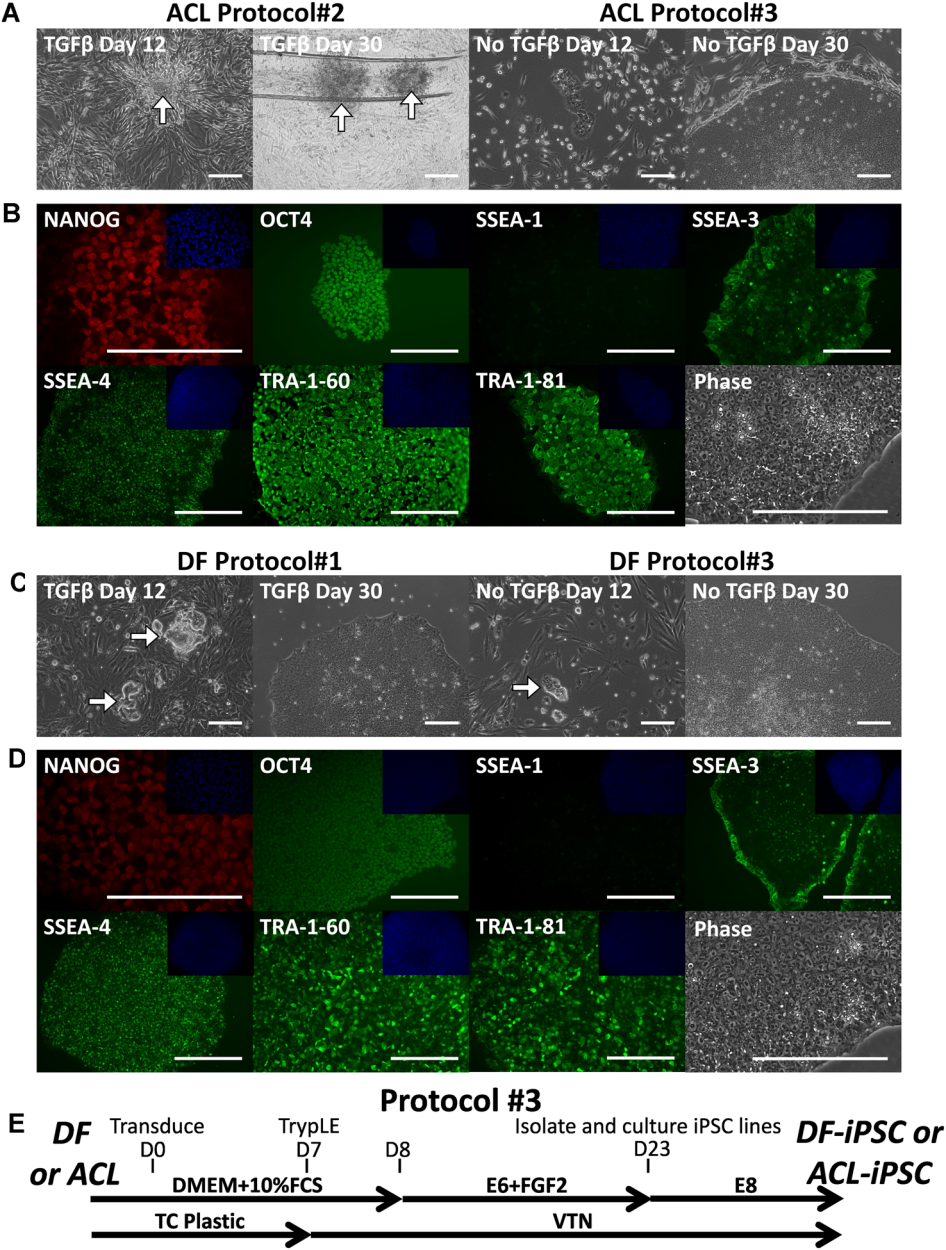
Reprogramming of anterior cruciate ligament (ACL) (ACL‐iPSC‐SW175A) and dermal fibroblast (DF) (DF‐iPSC‐SW174A) from a third donor in the presence (protocol#2/#1) or absence (protocol#3) of transforming growth factor β (TGF‐β). (A) Generation of ACL‐iPSCs in the presence (protocol#2) or absence (protocol#3) of TGF‐β, arrows indicate emerging colonies. With TGF‐β (protocol#2) emerging colonies grow on top of the ACL cells, scoring gives emerging colonies room to grow and prevents rolling up. Without TGF‐β (protocol#3) emerging colonies grow among ACL cells on the vitronectin (VTN) substrate and have more space to grow, scoring was not needed. (B) Passage 17 ACL‐iPSC‐SW175A line (generated using protocol#3) stained positive for antibodies to pluripotency‐associated markers and SSEA‐1, 4′,6‐diamidino‐2‐phenylindole (DAPI) nuclear staining shown in the top right quadrant. For ACL reprogramming in the presence of TGF‐β (protocol#2): one line was generated from 20,000 somatic cells, giving an efficiency of 0.005%. For ACL reprogramming in the absence of TGF‐β (protocol#3): eight lines were generated from 20,000 somatic cells, giving an efficiency of 0.040%. (C) Isogenic DF was also reprogrammed in parallel (DF‐iPSC‐SW174) in the presence (protocol#1) and absence (protocol#3) of TGF‐β. Emerging colonies grew on VTN substrate and have space to grow in both the presence or absence of TGFβ. (D) Passage 15 DF‐iPSC‐SW174A (generated using protocol#1) stained positive for antibodies to pluripotency‐associated markers and SSEA‐1, DAPI nuclear staining shown in the top right quadrant. For DF reprogramming in the presence of TGF‐β (protocol#1): 77 colonies with pluripotent morphology were observed from 20,000 somatic cells giving an estimated efficiency of 0.385%, 11 colonies were picked. For DF reprogramming in the absence of TGF‐β (protocol#3): 33 colonies with pluripotent morphology were observed from 20,000 somatic cells giving an estimated efficiency of 0.165%, four colonies were picked. (E) Schematic illustration of protocol#3, note the lack of TGFβ from day 8 until colony isolation and the lack of scoring on day 12. [Color figure can be viewed at wileyonlinelibrary.com]

As ACL and DF cells respond differently to TGF‐β (Fig. 4), we compared ACL cell reprogramming in the presence (E8, protocol#2) or absence (E6 + FGF2, protocol#3) of TGF‐β from a third individual. Consistent with the first two donors (ACL‐iPSC‐SW157 and ACL‐iPSC‐SW163), ACL‐iPSC colonies tended to grow on top of the ACL cells in the presence of TGF‐β (protocol#2) and could only be isolated from wells when the “scoring” technique was used (Fig. 5A). In the absence of TGF‐β (protocol#3), ACL cells proliferated less, allowing emerging iPSC colonies space to adhere to the VTN coated plastic (Fig. 5A), the colonies grew to a good size in the absence of TGF‐β and were easily isolated by manual cutting on day 30. Isolated ACL‐iPSC colonies were expanded to passage 15 using our standard iPSC culture; they displayed characteristic pluripotent cell morphology and were positive for pluripotent markers (ACL‐iPSC‐SW175 line; Fig. 5B). In contrast to ACL, there was no improvement in DF reprogramming in the absence of TGF‐β (DF‐iPSC‐SW174 line; Fig. 5C and D).

### DF‐iPSCs and ACL‐iPSCs Display Similar Marker Expression During Pluripotency and Early Differentiation

**Figure 6:**
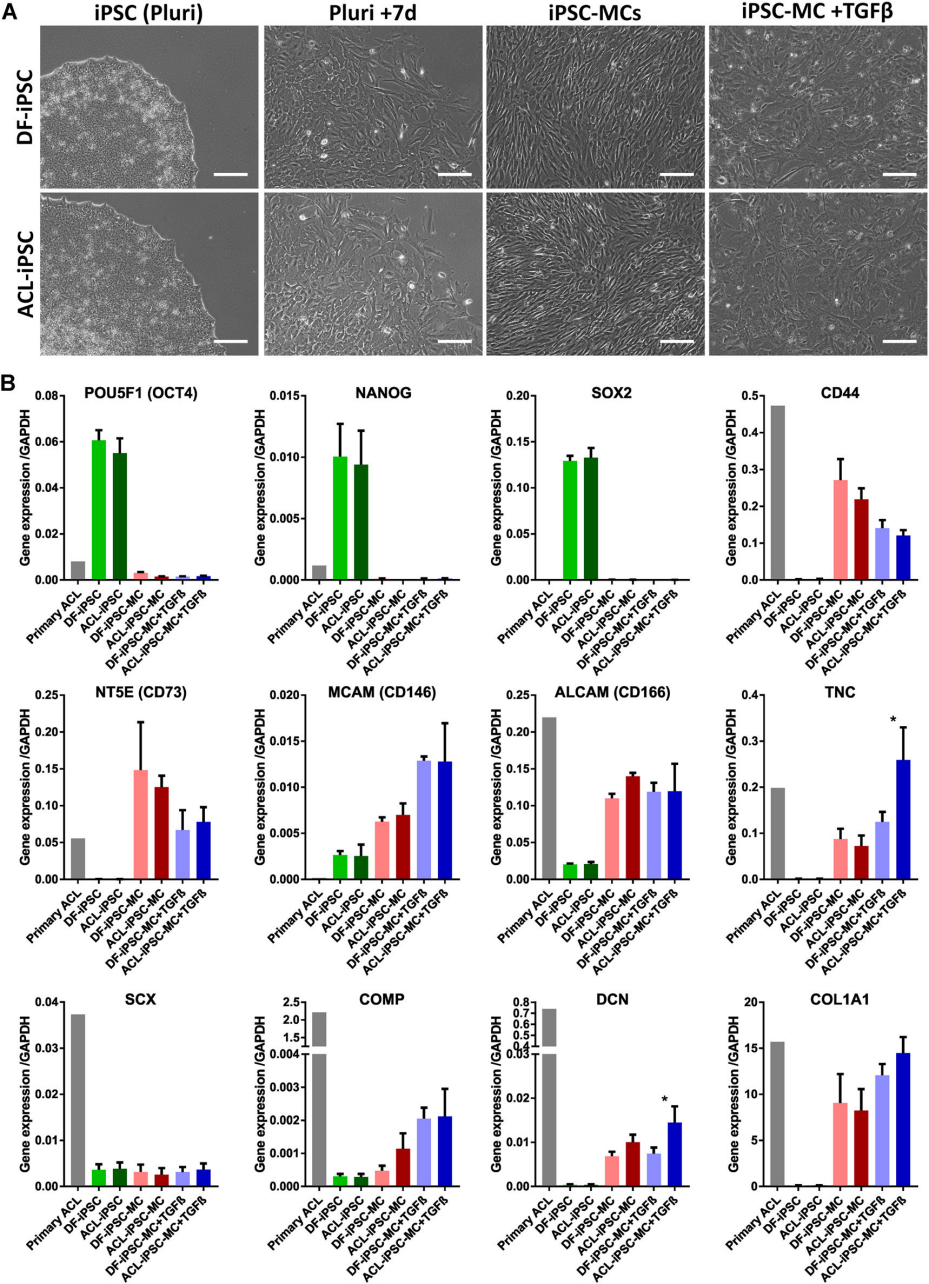
Dermal fibroblast‐induced pluripotent stem cells (DF‐iPSCs) and anterior cruciate ligament (ACL)‐iPSCs display similar marker expression during pluripotency and early differentiation. (A) Phase‐contrast images of DF‐iPSC and ACL‐iPSC differentiation to iPSC‐MC and subsequent iPSC‐MC stimulation with 10 ng/ml transforming growth factor β3 (TGF‐β3) for 7 days, scale bars represent 200 μm. (B) Quantitative real‐time polymerase chain reaction (qRT‐PCR) for pluripotent (POU5F1, NANOG, and SOX2), mesenchymal (CD44, NT5E, MCAM, and ALCAM) and ligament/tendon (TNC, SCX, COMP, DCN, and COL1A1) maker expression in primary (passage 3) ACL cells, DF‐iPSC (pluripotent), ACL‐iPSC (pluripotent), DF‐iPSC‐MCs, ACL‐iPSC‐MCs, DF‐iPSC‐MCs + TGF‐β and ACL‐iPSC‐MCs + TGF‐β. With the exception of primary (passage 3) ACL, data represented a minimum of four biological replicates from the two donors, including one pair of isogenic DF and ACL derived iPSC and iPSC‐MC lines. After removal of primary (passage 3) ACL data, statistical differences between DF and ACL derived cells were calculated using a one‐way analysis of variance (ANOVA), followed by Bonferonni multiple comparison adjustments (*p < 0.05). [Color figure can be viewed at wileyonlinelibrary.com]

To determine the differentiation ability of ACL‐iPSCs we generated mesenchymal cells from iPSCs (iPSC‐MCs) from both DF‐iPSCs and ACL‐iPSCs, using a protocol adapted from Nakagawa et al.^28^ Briefly E8 (pluripotent maintenance medium) was switched to MesenPRO RS™ (mesenchymal cell maintenance medium) causing altered cell morphology (Fig. 6A). After 7 days cells were passaged to gelatine coated plates, grown for a further 7 days and then passaged on tissue culture plastic thereafter. At this stage (iPSC‐MC) cells had gained a fibroblast‐like morphology, lost expression of pluripotency‐associated markers *POU5F1* (OCT4), *NANOG*, and *SOX2*, and gained expression of genes encoding mesenchymal markers *CD44, NT5E* (CD73), *MCAM* (CD146), and *ALCAM* (CD166) (Fig. 6B). The iPSC‐MCs also expressed more *TNC, DCN*, and *COL1A1* than pluripotent iPSCs (Fig. 6B). At the pluripotent and MC stages there were no significant differences between DF‐iPSCs and ACL‐iPSCs or between DF‐iPSC‐MCs and ACL‐iPSC‐MCs. As TGF‐β can enhance expression of tendon markers in mesenchymal cells,^29^ and primary ACL cells display a stronger response to TGF‐β then DF (Fig. 4), we hypothesized ACL‐iPSC‐MCs would display a stronger response to TGF‐β then DF‐iPSC‐MCs. Indeed, ACL‐iPSC‐MCs display higher expression of *TNC* (*p* = 0.028) and *DCN* (*p* = 0.032) than DF‐iPSC‐MCs when cultured in the presence of TGF‐β for 7 days. The expression of *SCX, COMP*, and *DCN* in iPSC‐MCs (both with and without TGF‐β) was lower than in primary (passage 3) ACL cells (Fig. 6B), indicating iPSC‐MCs require further differentiation to acquire a true ligament phenotype.

In summary, ACL‐derived iPSC lines were generated from three individuals; two with TGF‐β and “scoring” using protocol#2 (ACL‐iPSC‐SW157 and ACL‐iPSC‐SW163), and one without TGF‐β using protocol#3 (ACL‐iPSC‐SW175). For comparison, isogenic DF‐derived iPSC lines from two of these individuals (DF‐iPSC‐SW156 and DF‐iPSC‐SW174) were also generated. Protocol#3 (Fig. 5E) was the most efficient for reprogramming ACL cells.

## DISCUSSION

Here we report the conditions required for reprogramming of ACL cells to hiPSCs. ACL‐iPSCs may provide a useful pluripotent cell source for future ligament regenerative medicine therapies and for understanding human ligament development.

Ligaments give joints strength and stability and can withstand repeated cyclic loading. ACL rupture is a common injury that does not self‐repair.^30^ Current treatment usually involves reconstruction using autograft ligament from elsewhere in the body but leads to reduced movement.^31^ PSC‐differentiation to a ligament is emerging as a potential regenerative medicine treatment,^21^, ^22^ however there is no widely accepted cell source for such applications. Based upon the evidence for some epigenetic memory of iPSCs for their cell of origin,^15^, ^18^, ^19^, ^20^ ACL‐iPSCs would provide an ideal cell source for differentiation to the ligament, aimed at both understanding ligament development and potential for cell therapy. The generation of a ligament derived iPSC line has not previously been reported, yet iPSCs have been generated from other tissues that may be considered unusual or more difficult to reprogram, for example, human chondrocytes.^14^

This study is the first to successfully generate hiPSCs from the ACL and the first to generate hiPSCs from any true ligament (a ligament connecting bone to bone). These ACL‐iPSCs were generated using a feeder‐free protocol, displayed pluripotent morphology, pluripotent marker expression, normal karyotype and can differentiate to cells of all three germ layers. Previous studies have generated iPSCs from the periodontal ligament (connecting tooth to the bone), however, this was using mitotically inactivated mouse embryonic fibroblasts (MEFs) as feeders.^13^

During reprogramming ACL cells produced a stronger contraction response than DF cells to low concentrations of TGF‐β (2 ng/ml) (a concentration commonly found in PSC culture medium), which hindered PSC isolation. TGF‐β is known to play critical roles in differentiation and maintenance of the ligament, it causes the contraction of fibroblast cell lines^32^ and during equine ESC differentiation to tendon.^33^ Here we observed increased expression of *TNC, COMP*, and *COL1A1* in ACL cells in response to TGF‐β, suggesting reinforcement of the tendon/ligament phenotype, which hindered reprogramming. Interestingly, our data indicate TGF‐β may act in synergy with FGF2 to increase ACL cell proliferation, hindering reprogramming further. TGF‐β is also required for PSC maintenance^34^, ^35^, ^36^ and is a component of the widely used chemically defined E8 stem cell medium.^37^

TGF‐β ligands and downstream signaling are important considerations during reprogramming, for example, in determining cell fate during reprogramming of human urine cells,^38^ and in mouse where inhibition of TGF‐β can replace the need for SOX2 during reprogramming of embryonic fibroblasts.^39^ In addition TGF‐β is known to inhibit mesenchymal‐to‐epithelial transition, a process required for the early stages of reprogramming.^40^ Indeed, TGF‐β has been shown to be dispensable for reprogramming of DFs,^37^, ^41^ as also indicated in our study, and in some studies its inhibition was reported to enhance reprogramming.^42^ We show ACL reprogramming is more efficient in the absence of TGF‐β, although this is still relatively inefficient compared with DF reprogramming, suggesting further refinement of the ACL reprogramming protocol is possible. After the 23 days of the reprogramming protocol, the routine E8 medium (including TGF‐β) was reapplied to ensure the maintenance of the emerging iPSC colonies, which then behaved in a way that was indistinguishable to those derived from DF.

Epigenetic changes occur during the reprogramming of somatic cells to iPSCs,^43^, ^44^ however, this process is incomplete, meaning iPSCs may retain an epigenetic memory of their somatic origin,^15^, ^18^, ^19^, ^20^ which can result in altered chromatin structure, DNA methylation, gene expression and biased differentiation, skewed towards the tissue of origin or related tissues. As expected, *TNC, SCX, COMP, DCN*, and *COL1A1* transcripts were expressed in negligible amounts in iPSCs from both DF and ACL. Tendon/ligament markers were increased in iPSC‐MCs compared with iPSCs, although none of them reached the expression level observed in primary (passage 3) ACL cells. While at the MC stage there was no difference in expression of these transcripts between DF and ACL, following TGF‐β treatment higher *TNC* and *DCN* expression was detected in ACL‐iPSC‐MCs than DF‐iPSC‐MCs. Therefore although cells were not fully differentiated to tendon/ligament, differences indicative of a tendency for ACL‐iPSC‐MCs to better form tendon/ligament than DF‐iPSC‐MCs were observed. Furthermore, although *TNC, SCX, COMP, DCN*, and *COL1A1* are expressed in cultured ACL cells, they are also expressed in cultured DFs and therefore unlikely to be the best discriminators of DF‐iPSCs and ACL‐iPSCs. DF‐iPSC and ACL‐iPSC differentiation to mature tendon/ligament constructs would be required to determine if ACL‐iPSCs retain an epigenetic memory that is functionally important, as previously suggested for other cell types. In mouse, iPSCs derived from early hepatoblasts are more easily differentiated to a hepatic lineage than MEF‐iPSCs or mESCs.^18^ In human, cells differentiated from pancreatic islet beta cell‐derived iPSCs have been reported able to secrete more insulin than iPSCs derived from other isogenic tissues.^9^

Here we report the conditions required to generate iPSCs from the ligament. We provide two alternative protocols, both of which disrupt the three‐dimensional structure produced by ACL cell sheets, protocol#2 does this mechanically, while protocol#3 does this through removal of TGF‐β. Manual mechanical disruption of the ACL cell sheet within protocol#2 is a source of variation and thus a limitation. Protocol#3 does not involve this manual input from the user and is a more robust and reliable means of generating iPSCs. Such ACL‐iPSCs are likely to provide an ideal pluripotent cell source for ligament differentiation, both to further understand human ligament development and for tissue engineering applications. This report extends the number of iPSC cell sources and will advance skeletal tissue regenerative medicine.

## AUTHORS’ CONTRIBUTION

S.W., F.S.I., T.E.H., and S.J.K. contributed to study conception and design. F.S.I. provided the study materials. S.W., N.B., and S.L.D. performed experimental procedures and/or collection of data. S.W., N.B., and S.J.K. performed data analysis and interpretation. S.W. and S.J.K. wrote the paper. All authors read and approved the final manuscript. All authors gave consent for publication.
